# NeedFull – a Tweet Analysis Platform to Study Human Needs During the COVID-19 Pandemic in New York State

**DOI:** 10.1109/ACCESS.2020.3011123

**Published:** 2020-07-22

**Authors:** Zijian Long, Rajwa Alharthi, Abdulmotaleb El Saddik

**Affiliations:** Multimedia Communications Research LaboratoryUniversity of Ottawa 6363 Ottawa ON K1N 6N5 Canada; Department of Computer ScienceTaif University 125895 Taif 26571 Saudi Arabia

**Keywords:** Human needs analysis platform, social media, human needs detection, smart city, machine learning, big data, pandemic, twitter

## Abstract

Governments and municipalities need to understand their citizens’ psychological needs in critical times and dangerous situations. COVID-19 brings lots of challenges to deal with. We propose NeedFull, an interactive and scalable tweet analysis platform, to help governments and municipalities to understand residents’ real psychological needs during those periods. The platform mainly consists of four parts: data collection module, data storage module, data analysis module and data visualization module. The four parts interact with each other and provide users with a thorough human needs analysis based on their queries. We employed the proposed platform to investigate the reaction of people in New York State to the ongoing worldwide COVID-19 pandemic.

## I. Introduction

Nowadays social media is changing people’s life [1]. People can share news and opinions and interact with each other constantly on the Internet anytime anywhere with the rapid development of Social Network Service (SNS) [2]. People post large numbers of spatial and temporal-based data including texts, images and videos on popular social media platforms such as Twitter, Facebook, and Instagram leading to the rise of social big data. For example, people on Twitter post around 6000 tweets each second about a vast range of events happening all over the world.^1^^1^https://about.twitter.com/company

Since social media is becoming a more and more important part of people’s daily life [3], exploring and mining social big data to understand people’s various affective states is significant and meaningful. A simple way of analyzing human affective states is identifying the sentiment polarity (positive, neutral and negative). Some related works are shown as follows. In 2009, Alec Go *et al.* first used machine learning methods to automatically conduct sentiment analysis on the text on Twitter, and added emoticons to the system, which greatly improved the accuracy of the system [4]. Asli Celikyilmaz *et al.* employed a probabilistic model to classify the text on twitter into polar(tweets with positive or negative sentiment) and non-polar(tweets without sentiments), and then used emotional vocabulary to classify the emotional polarity of polar tweets [5]. Human need detection, which goes deeper than sentiment analysis, can recognize the psychological need type (relatedness, competence and autonomy) according to the Human Needs Theories (HNT) [6] and discover if this need is satisfied or not. The awareness of human needs is a significant step to improve people’s well-being. However, it did not receive much attention yet. In [7], the authors propose a framework with multiple layers to recognize human needs during critical events based on data from Twitter.

Based on the above works, some social media analysis platforms have been constructed. Li *et al.* proposed City Digital Pulse (CDP), a cloud-based heterogeneous data analysis platform. The system collects geo-tagged data from Twitter and Instagram and conducts sentiment analysis on these data [8]. In 2019, Daniel *et al.* presented a multimodal affect and context sensing platform which is designed to enable easy prototyping of novel computer interfaces that sense, respond, and adapt to human emotion [9]. To the best of our knowledge, there is no human psychological needs analysis platform based on social media. Therefore, we propose an interactive and scalable human needs analysis platform to solve this problem.

The remainder of the paper is organized as follows. In section 2, we present a review of related work of detecting human needs. System design and implementation of our NeedFull platform are illustrated in section 3. In section 4, We investigate the public reaction to the ongoing worldwide COVID-19 pandemic in New York State using our system. In the end, section 5 concludes our work and discusses future work.

## II. Related Work

Generally speaking, analyzing human affective states involves three steps: sentiment analysis, emotion detection, and human need detection. The first step is sentiment analysis, also known as opinion extraction, is the computational treatment of opinions, sentiments and subjectivity of text are employed [10]. Sentiment analysis techniques can be divided into three categories based on different levels:

1) Document-level sentiment analysis aims to automate the task of classifying a textual review, which is given on a single topic, as expressing a positive or negative sentiment [11]. Mathews *et al.* proposed a lexicon-based method to perform polarity calculation on the multilingual dataset which consists of a mix of reviews in English and Malayalam for sentiment [12]. The proposed methodology treats both types of lexicons differently and it gives more accurate results for sentiment analysis. To solve the problem that the existing researches cannot exploit the deep semantic information of documents, Liu *et al.* proposed a novel hierarchical neural network model based on dynamic word embeddings (HieNN-DWE) for document-level sentiment classification [13]. The model consists of two layers: the first one uses bidirectional gated recurrent unit (BiGRU) and attention mechanism to encode sentences and in the second layer, both BiGRU and convolutional neural network (CNN) are employed to capture features in the sentences. This model outperforms existing methods on four public datasets for document-level sentiment classification.
2) Sentence-level sentiment analysis refers to the process of identifying the sentiment polarity of a single sentence. Many feature-based statistical methods, such as Naive Bayes (NB), Random Forest (RF) and Support Vector Machine (SVM) were employed to solve this problem and achieved high accuracy in classifying sentiment type in the past 20 years. Recently, models based on deep learning methods have made progress in this area. In 2019, Shen *et al.* proposed a model which combines a Bidirectional Encoder Representation from Transformers (BERT) with BiGRU to gain the contextualized embeddings before performing sentiment analysis [14]. Some researchers also believe that the ensemble of deep learning methods and traditional feature-based methods could further improve the accuracy of sentiment classification. Zhang *et al.* proposed an ensemble method which uses support vector machine with naive bayes features (NB-SVM), an enhanced model to optimize CNN [15].
3) Aspect-level sentiment analysis is designed to solve the problem when multiple aspects show up in a complex sentence which is quite common in the real world. Different from the other two categories of methods, aspect-level sentiment analysis needs to discover all the aspects involved in the text first and then perform sentiment analysis for each aspect. Wang *et al.* proposed the aspect-level sentiment capsules model (AS-Capsules), which could perform aspect detection and sentiment classification at the same time [16]. Moreover, they added the attention mechanism to find out aspect related words and sentiment words without any linguistic knowledge. In 2020, Lu *et al.* proposed an interactive rule attention network (IRAN) considering the influence of grammatical rules [17]. IRAN simulates the grammatical functions at the sentence and also uses an attention network to learn attention information from context.

Meanwhile, textual emotion detection, whose task is to classify a text into one or more predefined emotion categories by extracting the emotional elements in the textual content, has gained a lot of attention in recent years. The emotional classification methods mainly include dictionary-based methods, rule-based methods, machine learning-based methods, composite methods, and multi-label methods [18]. Chatterjee *et al.* gathered large scale data from social media and proposed a novel Deep Learning based approach to detect emotions including happy, sad, and angry in texts [19]. Zhong *et al.* proposed a Knowledge-Enriched Transformer (KET) to understand human emotions by using a context-aware affective graph attention mechanism [20]. However, as the last step of analyzing human affective states, there are only a few works about detecting human needs. In [21], the authors proposed a multilayered psychological-based reference model to assess citizen needs during any event at any time. In fact, the human needs detection model we employed in our platform is based on this work.

## III. NeedFull - a Human Needs Analysis Platform

### A. System Architecture

**FIGURE 1.**
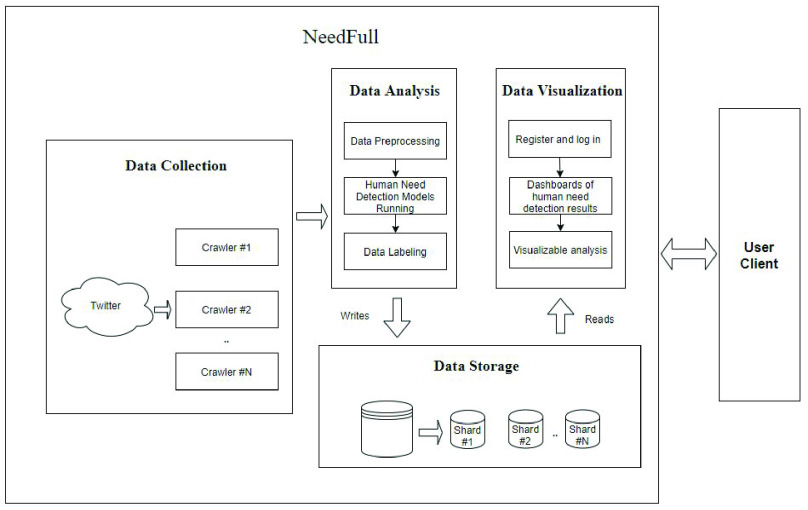
Architecture of NeedFull platform.

As shown in Figure 1, our proposed human needs analysis platform consists of four main parts: data collection module, data storage module, data analysis module and data visualization module. The whole process of how NeedFull works is illustrated as follows: the crawlers in the data collection module gather raw data from Twitter. Then our human need detection models in the data analysis module will label the data before the data is put into the database. When a user enters a query through the user interface, they will get all the related items in the database by the index system of the data storage module and a comprehensive human needs analysis of these tweets is then presented and depicted in the data visualization module.

### B. Data Collection

**FIGURE 2.**
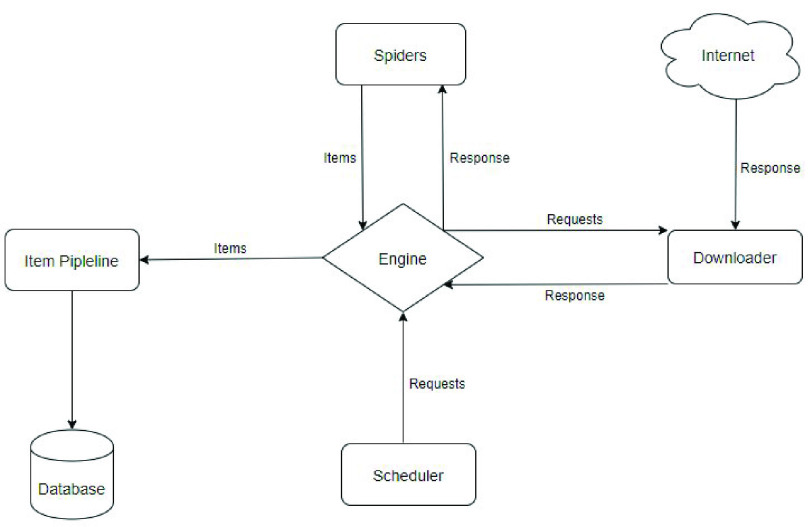
Architecture of NeedFull crawler for historical data.

Since the standard Search API of Twitter allows developers to collect tweets published in the past 7 days, we made our own crawler based on it to collect recent data. However, Twitter does not provide developers with an API to download historical data. Therefore, we designed a historical data crawler as a supplement in case old data is required. The crawler is built on Scrapy, an open-source web-crawling framework^2^ and the architecture of our NeedFull crawler for historical data is shown in Figure 2: The Engine gets arranged requests from the Scheduler and sends the requests to the Downloader; Once finishing downloading the web page from the Internet, the Downloader generates a response with that page and sends it to the Spider through the Engine for processing; Then the scraped items are passed to the Item Pipelines where we can specify the structures of these items before stored into the database.^2^https://scrapy.org/

### C. Data Storage

**FIGURE 3.**
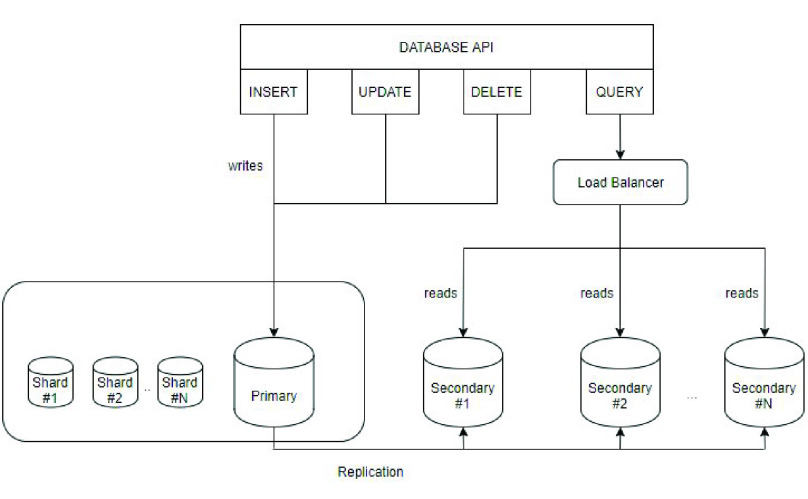
Design of database in NeedFull platform.

We built our database based on Mongodb, a dynamic and scalable NoSQL database as it can provide high performance when processing Big Data. As our platform needs to support a small number of write operations but a large number of read operations, we built a replica set in Mongodb for the original database which has a primary node and multiple secondary nodes to scale out read operations. As Figure 3 shows, write operations such as inserting new data, updating or deleting previous data go to the primary database and read operations like querying go to the secondary databases. Since a primary database could have multiple secondary databases, we could add more secondary databases when the number of the data grows larger and the load balancer can easily find the relatively free one. This also makes the system easy to scale. Moreover, these databases can be synchronized by replicating the log in the primary database once there are new write operations in the primary database. Even when the primary database breaks down, we can still use these backup databases to keep the system running which significantly improves the security and robustness of our platform. Since the cost of performing a read operation in a single table with all the data is extremely high when the number of data grows extremely large, we partitioned the original table by building a table for each day of each city and created an index for every table. When a query with specified dates and cities enters our system, the load balancer can first allocate it to a relatively free secondary database node. Then the index will find the corresponding ids of items related to the query. Therefore, all the related items can be extracted fast.

### D. Data Analysis

**FIGURE 4.**
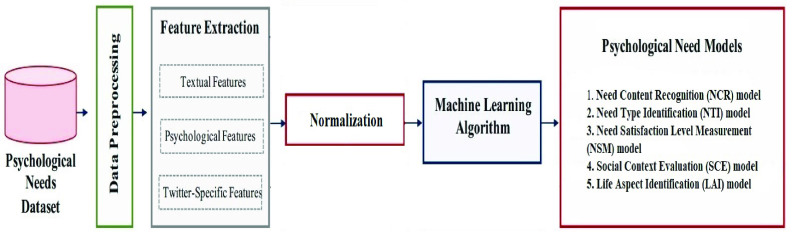
Offline training and testing phase of the human need detection framework based on [7].

**FIGURE 5.**
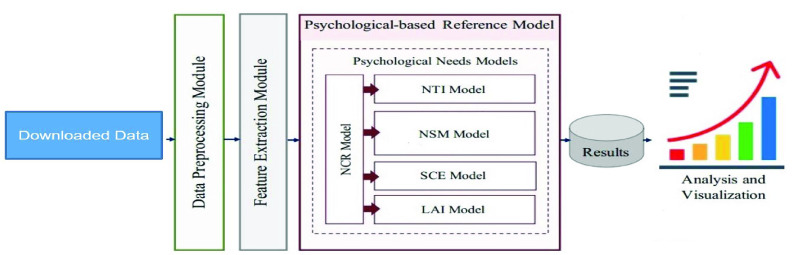
Online human need detection and analysis phase of the human need detection framework based on [22].

We employed a human need-detection framework introduced in [22]. The framework incorporates an offline phase to design and develop the need models following the methodology presented in Figure 4 and an online phase that we used for automatically recognizing human needs in real time as explained in Figure 5. We utilized the following human needs models: Need Content Recognition (NCR) model, Need Type Identification (NTI) model, Need Satisfaction Level Measurement (NSM) model, Social Context Evaluation (SCE) model, and Life Aspect Identification (LAI) model. The meaning of each model is explained as follows: Layer 1 (NCR model) is designed to recognize need content in the post. If need content exists, the post goes to Layer 2 (NTI model) and Layer 3 (NSM model) to identify the need type (relatedness, competence, and autonomy) and its satisfaction level (satisfied, dissatisfied, and neutral). Layer 4 (SCE model) is constructed to assess the quality of an individual’s social context (supportive, unsupportive, and not clear), and Layer 5 (LAI model) identifies the aspect of life involved in the need experience. After collecting data of interest from Twitter, we ran the required need models to obtain individuals’ psychological need aspects as well as analyze and visualize them.

#### 1) Data Preprocessing

The tweets we downloaded from Twitter are stored in a JavaScript Object Notation (JSON) format, where each tweet consists of dozens of key-value pairs. As we focused on analyzing only the textual content, we obtained all the texts using the key “text.” In the online phase, we followed the same offline data preprocessing steps presented in [7]. We kept all stop words (e.g., “a,” “an”) because they are meaningful and influence the results. Words derived from the same word (e.g., “plays,” “playing,” “player”) can be replaced by their original word (i.e., “play”). Therefore, we employed “stem,” another library from NLTK, to find these words and remove their suffixes. Because the tweets are full of emojis and emoticons widely used by people to express their feelings, we kept all emojis and emoticons provided by Twitter and considered them as a part of the texts. The last step was to tokenize the tweets by dividing the texts into separate words.

#### 2) Feature Extraction

Feature extraction is the process of removing a list of words from the text data and then transforming them into a feature set usable by a classifier. In other words, a tweet needs to be converted to a vector that can represent the tweet. From each tweet, we extracted the text-based features, psychological features, and Twitter-specific features developed during the offline phase [7]. Text-based features include the Bag-of-Words model (BoW) and Ngram Language Model (LM) extracted using the Term Frequency-Inverse Document Frequency (TF-IDF) weighting scheme. For psychologically related aspects, we extracted features included in the Linguistic Inquiry and Word Count (LIWC) model and Linguistic Category Model (LCM). For Twitter-specific features, including emojis and hashtags, features were extracted by counting the number of hashtags and emojis (i.e., categories and colors of emojis).

#### 3) Normalization

Because the vectors that we constructed to represent tweets are composed of features from text-based, psychological, and Twitter-specific three different aspects, the values in vectors have various ranges. Features with bigger ranges may have a bigger influence on the classification result. To ensure that we capture the accurate information, we employed Min-Max scaling:

\begin{equation*} x_{new}=\dfrac {x - x_{min}}{x_{max} - x_{min}}\end{equation*}

where $x_{new}$ is the normalized value for x and it is in the range of [0, 1].

#### 4) Training and Evaluation of the Models

For the human need classification models developed in [22], we employed the Support Vector Machine (SVM)-based models, which offer very high accuracy and speed compared to other classifiers, such as logistic regression, decision trees, Multinomial Naive Bayes algorithm, and Random Forest algorithm. Table 1 shows the Recall, Precision, $F_{score}$, and Accuracy of the SVM-based models in each layer of the human need framework.

**TABLE 1:** Accuracy, Recall (R), Precision (P) and $F_{score}$ for the Human Need Models Based on [22]

Psychological need recognition models	Accuracy %	R	P	F
Need Content Recognition (NCR) Model	79.13	0.791	0.82	0.77
Need Type Identification (NTI) Model	81.97	0.81	0.82	0.817
Need Satisfaction level Measurement (NSM) Model	93.56	0.93	0.93	0.93
Social Context Evaluation (SCE) Model	75.70	0.75	0.76	0.75
Life Aspect Identification (LAI) Model	60.48	0.59	0.60	0.59

### E. Data Visualization

**FIGURE 6.**
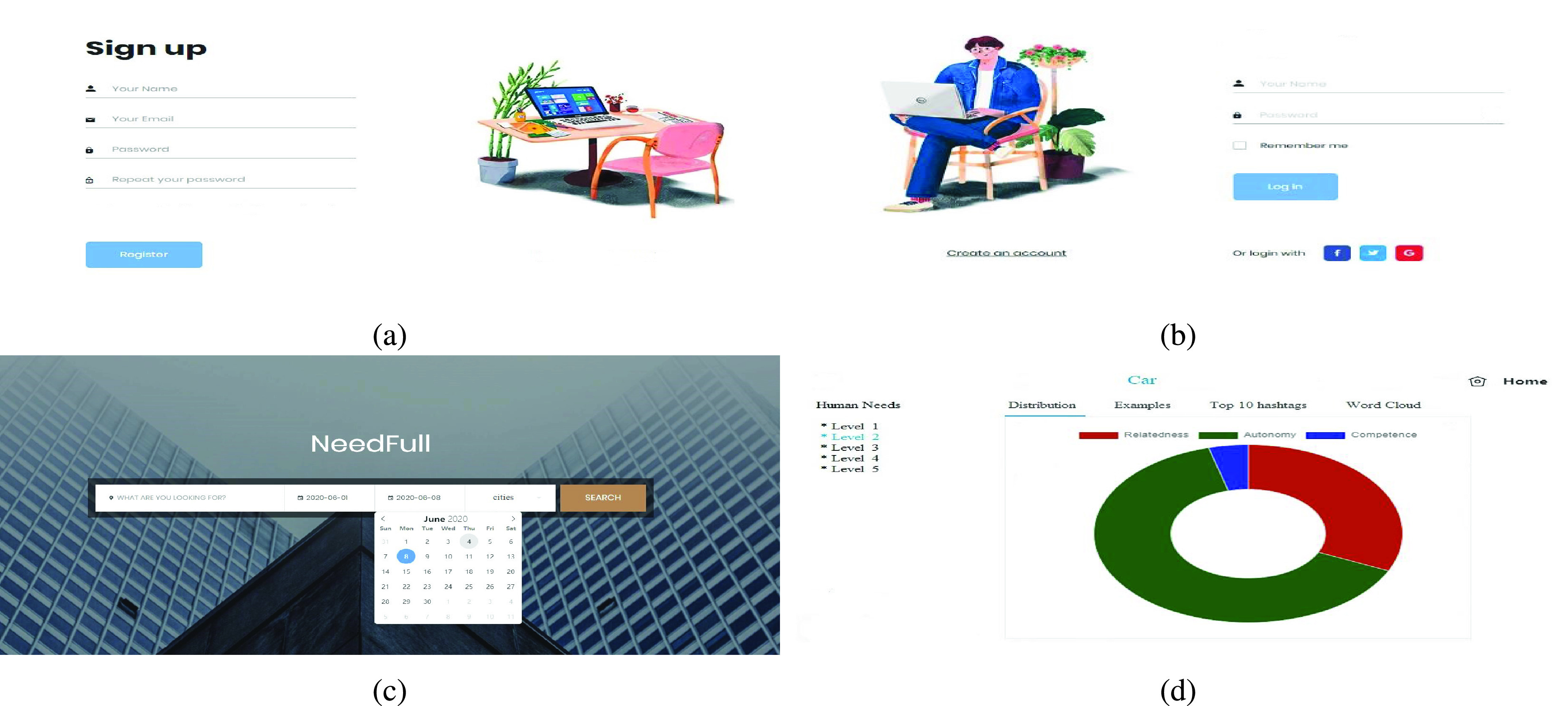
User interface of NeedFull: Register Page (a), Login Page (b), Search Page (c), and Result Page (d).

We designed a user-friendly interface to interact with users and visualize the analysis result. The interface is built on Chart.js which is a free open-source JavaScript library for data visualization. Users need to register for accounts on the register page (Figure 6a) first and log in with their accounts on the login page (Figure 6b). Once a query of interest with keywords, dates, and locations is specified by users on the search page (Figure 6c), they will enter the result page (Figure 6d) with a thorough human needs analysis. On the result page, it provides interactive dashboards of human needs distribution. More details will be demonstrated in the next section with the analysis of the public reaction to the COVID-19 pandemic in New York State.

## IV. Human Needs Analysis of the COVID-19 Pandemic in New York State

### A. Experimental Design

We inspected the reaction of people in New York State to the ongoing worldwide COVID-19 pandemic. The first case of COVID-19 in New York was confirmed on March 1. As of April 6, 2020, there have been over 130000 confirmed cases in the state, and of those 4159 people have died with the highest number of confirmed cases of any state in the United States.^3^ To control the spread of the coronavirus, the state has ordered nonessential businesses to shut down, banned gatherings of any size and mandated that people stay six feet away from each other. People’s daily life has been extremely influenced by the outbreak of the coronavirus which also leads to the tremendous amount of posts related to COVID-19 in social media platforms. To obtain as many related tweets as possible from New York State, we used the hashtags such as #coronavirus, #COVID-19, #outbreak, #pandemic and #virus and set the search area in New York State in our data collection module. Therefore, we ended up with a total of 154486 publicly accessible English tweets from March 1 to April 5. We believe ongoing social topics play an important role in the changes of people’s psychological needs. Therefore, we used the term frequency (TF) measurement to acquire the top 10 frequent hashtags for every week as hashtags are mainly used to denote specific topics of conversation on Twitter. Moreover, we studied the most frequently used words regarding coronavirus and present them in word clouds where the size of a word shows how important it is in the discussion.^3^https://www.cdc.gov/

### B. Human Needs Analysis of the COVID-19 Pandemic in New York State for Each Week

Figure 8 illustrates that in week 1, the first week when the first coronavirus case was confirmed, over half of the tweets expressed frustration for each psychological need: relatedness (60.32%), autonomy (50.67%), competence (57.26%). As shown in Table 2, people began to talk about it using the hashtags #CoronaOutbreak, #CoronavirusUSA, #CoronavirusChina (the first country where the corona virus outbreaks) and #CoronaVirusUpdate. Some measures proposed by the Centers for Disease Control and Prevention (CDC) and the World Health Organization (WHO) used to control the spread of the virus such as #WashYourHands and #PayAttention also caused a broad discussion. From the word cloud (a) in Figure 9, we can see that words like “CDC”, “flu”, “hand”, “Trump”, “work” and “case” appear in a huge amount of tweets. Although there were not many confirmed cases by then, people started to worry about the outbreak in New York State since it has killed thousands of lives and extremely damaged the economy in China. The worries lowered the satisfaction level for each psychological need. Here are some examples collected from week 1:

• I feel like this a great time to identify 1 or 2 people as your primary partners until this Corona Virus dies down. – Satisfied relatedness need
• I won’t be hanging my white friends till Corona virus over.-Dissatisfied relatedness need
• Yeah I get it, the flu has killed waaaay more people than the Corona Virus. But as a mother to young children I AM going to stress about the virus the same way I stress about the flu. If it has the possibility to KILL my children, I’m gonna worry. So shut the fuck up about it.-Satisfied autonomy need
• I think I got the corona virus and need to go home to isolate myself.-Dissatisfied autonomy need
• I have a better chance of listening to country on my own than getting the corona virus.-Satisfied competence need
• Why would we expect Trump and the GOP to do anything to protect our health from the corona virus or anything else when they have already depleted the EPA? They have rolled back 95 EPA protections endangering the environment and American lives.-Dissatisfied competence need

**TABLE 2:** Top 10 Most Frequently Used Hashtags in Tweets During the COVID-19 Pandemic in New York State Using NeedFull Platform

WEEK 1	WEEK 2	WEEK 3	WEEK 4	WEEK 5
#CoronaOutbreak	#CoronavirusPandemic	#pandemic	#pandemic	#CoronaVirusOutbreak
#CoronavirusUSA	#CoronaVirusUpdate	#nylockdown	#batflu	#pandemic
#CoronaVirusUpdate	#coronapocalypse	#CoronavirusPandemic	#Wuhan	#Trumpandemic
#WashYourHands	#outbreak	#socialdistancing	#CoronaVirusOutbreak	#Survive
#FakeNews	#CancelEverything	#ToiletPaperPanic	#CoronaVirusChallenge	#CoronaVirusChallenge
#CoronavirusChina	#NBAsuspended	#CoronavirusOutbreak	#China	#Quarantine
#Business	#SocialDistancing	#lockdown	#coronavirusupdates	#TrumpVirus
#CoronaVirusChallenge	#washyourhands	#CoronaCrisis	#LockdownNow	#stayhome
#PayAttention	#StockMarket	#ToiletPaperApocalypse	#Quarantine	#socialdistancing
#outbreak	#pandemic	#StayHome	#socialdistancing	#StayAtHomeSaveLives

**FIGURE 7.**
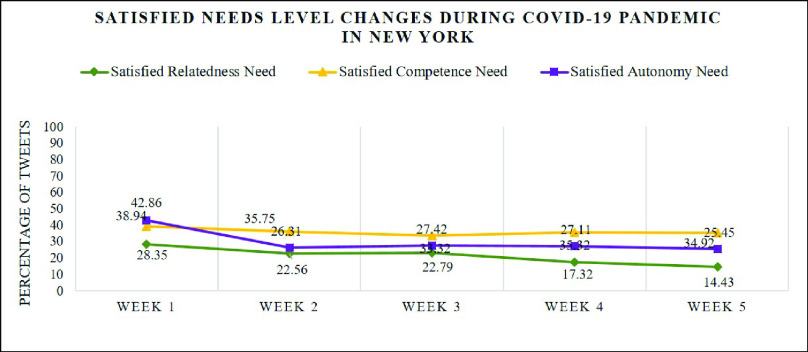
Measuring weekly change of satisfaction level during the COVID-19 pandemic in New York State using NeedFull platform.

**FIGURE 8.**
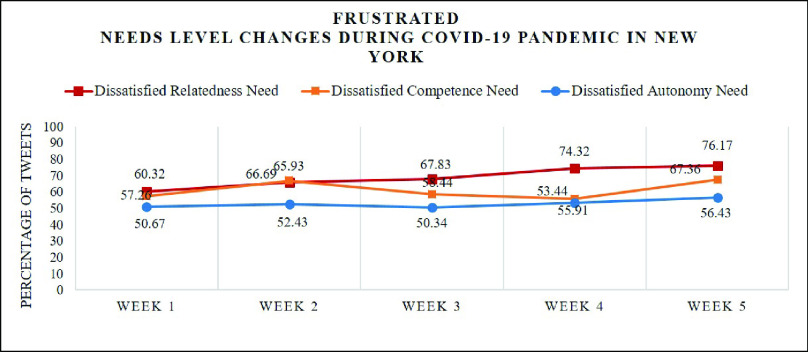
Measuring weekly change of frustration level during the COVID-19 pandemic in New York State using NeedFull platform.

**FIGURE 9.**
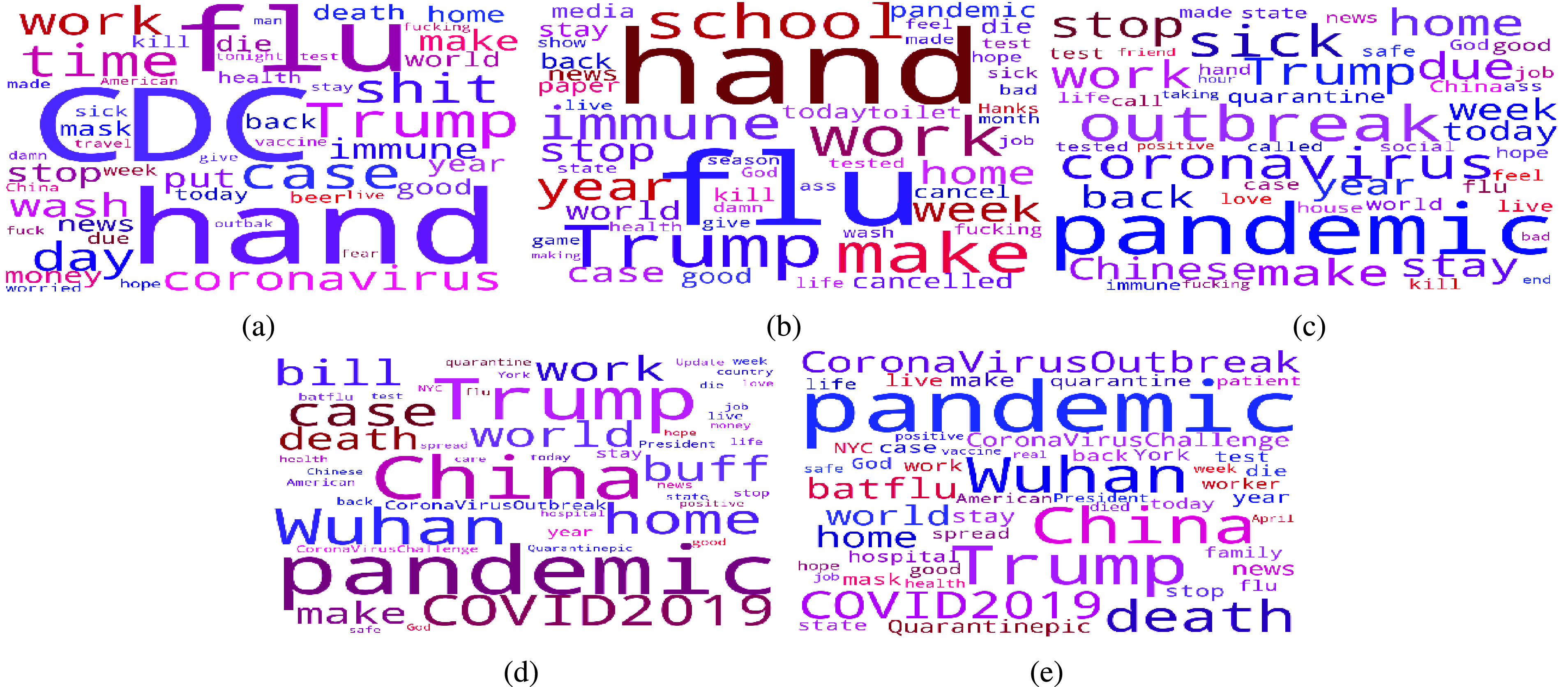
Word cloud generated from tweets during the COVID-19 pandemic in New York State using NeedFull platform: WEEK 1 (a), WEEK 2 (b), WEEK 3 (c), WEEK 4 (d) and WEEK 5 (e).

In week 2, as can be seen from Figure 7, there is a noticeable reduction of satisfaction of residents’ need. Compared to week 2, the satisfied relatedness need, autonomy need and competence need declined from 28.35% to 22.56%, 42.86% to 26.31%, and 38.94% to 26.31% respectively. Hashtags like #CoronavirusPandemic, #outbreak and #pandemic were widely used by residents in New York State shown in Table 2 as WHO has declared the coronavirus outbreak a pandemic on March 11. Protection measures such as #CancelEverything, #SocialDistancing and #washyourhands are still the center of topics since people realized the outbreak of coronavirus had begun in New York State. This can also been seen from the word cloud (b) in Figure 9 where. We can also find that some people pay much attention to the stock market and the National Basketball Association (NBA) using hashtags #StockMarket and #NBAsuspended. From online news, we know that, during this week, the coronavirus pandemic has had far-reaching consequences beyond the spread of the disease and efforts to quarantine it such as the crashed stock market and the suspended NBA season. We also noticed that the word “Hanks” showed up in the word cloud (b) in Figure 9. This is because the famous American actor Tom Hanks confirmed he and his wife had been diagnosed with coronavirus on March 11 which has aroused widespread concern. The impact of the coronavirus on people’s lives was gradually increasing and people shared their feelings and worries about it. Some examples are listed as follows:

• BREAKING NEWS: Peter Griffin has tested positive for Corona Virus. Send prayers for him and his family. – Satisfied relatedness need
• I’m actually terrified for my kids to get this corona virus going around! Especially my baby because she’s so young! -Dissatisfied relatedness need
• Can’t wait to see what happens after the corona virus pandemic is over. -Satisfied autonomy need
• Looks like I may have lost my job thanks to the corona virus. -Dissatisfied autonomy need
• Can’t believe stony brook is switching to all online classes in preparation for the corona virus that’s so crazy but smart. -Satisfied competence need
• We do not have access to Corona tests, these numbers are not accurate. Not only is prevention and treatment the key to this PANDEMIC (worldwide) but knowledge is power. You are failing our children and our city. #besmart. -Dissatisfied competence need

In week 3, there is no noticeable change of frustration and satisfaction level for each need with 67.83% of the relatedness, 58.44% of the autonomy and 50.34% of the competence being dissatisfied and 22.79% of the relatedness, 27.42% of the autonomy and 33.32% of the competence being satisfied shown in Figure 7 and Figure 8. It is easy to see that some new topics appear like #nylockdown and #lockdown from Table 2 as New York Governor Andrew Cuomo announced a strict lockdown including shutting down nonessential businesses and ordering all 40 million state residents to stay at home on March 20. These measures to battle the spread of the COVID-19 have caused broad panic among residents and made them rush to stock up essential items such as toile paper, canned food, water bottles and pasta. This also explains the appearance of the hashtags #ToiletPaperPanic and #ToiletPaperApocalypse in Table 2. We expect these lockdown measures would bring a distinguishable increase in frustration level. However, deeper analysis shows frustration level did not change that much. It seems buying essential items and getting ready for staying at home for a long period eased people’s anxiety in a way. Examples are posted in week 3:

• can’t wait for my kid to come home one day and show me he’s reading “ I Survived: The Corona Pandemic of 2020”. – Satisfied relatedness need
• My best friend can’t even come to my “closest friends only” baby shower because of the stupid corona virus. -Dissatisfied relatedness need
• after the corona virus calms down, i will start living my best life with or without the attendance of others. -Satisfied autonomy need
• So if this corona virus thing keeps getting out of hand. I’ll be quitting my job and I’m going quarantine myself at James Franco’s house. -Dissatisfied autonomy need
• I told myself I wasn’t going to go out this weekend. Thanks corona virus for helping me accomplish my goals. -Satisfied competence need
• i’m so over this corona virus. i just want to participate in my extracurricular and then graduate. every other high schooler except c/o 2020 had that chance. what did we do:(-Dissatisfied competence need

From Figure 8, we can see that, in week 4, there is a slight increase in frustration level for each need compared to week 3: relatedness (74.32%), autonomy (53.44%) and competence (55.91%). As Table 2 shows, people began to use some new hashtags such as #batflu which is considered by some people as how the first case in China got infected, #Wuhan (the first city where the outbreak of coronavirus happened) and #China. The word cloud (d) in Figure 9 also demonstrates that the most frequently used words are “China”, “Wuhan”, “Chinese” and “pandemic” as president of the United State Donald Trump kept using “Chinese Virus” instead of coronavirus or COVID-19 which connected the virus with China. However, the sustainable growth of frustration level denotes President Trump’s comments did not satisfy people’s psychological need though people talked more about China. Here are some tweet examples:

• Wonderful gesture James! Hollywood are you listening? Now would be a great time to show your appreciation for your success by publicly donating to a cause to defeat Corona Virus! I might even go to one of your movies. – Satisfied relatedness need
• Phew! even Chinese are not shopping here in New York their own people why are people are so judgmental and look at every Chinese as if they have corona virus which is not true! I have a lot of Chinese friends and so what? – Dissatisfied relatedness need
• Putting a little more time into cooking now that I am home more often during the Corona Virus Pandemic. These are photographs of me and my healthy lunch today. Remember good nutrition in the mist of staying home (avoid unhealthy snacks)! -Satisfied autonomy need
• Corona virus is so trash…. I was supposed to start swimming classes this weekend. -Dissatisfied autonomy need
• My move in date for my first apartment keeps getting pushed back…I’m about to find a cure for this corona virus myself. -Satisfied competence need
• I have lost my job a week before the corona virus shut down and applied for unemployment and was denied immediately. I then found a new job being an assistant teacher at a daycare but once the mayor ordered for schools to close then that meant my job closed so I couldn’t start. -Dissatisfied competence need

As the number of confirmed cases of coronavirus in New York State had gotten to over 130000 with 4159 people died in week 5, satisfaction levels, shown in Figure 7, reached the lowest so far where the percentages of satisfied relatedness, autonomy and competence are 14.43%, 25.45% and 34.92% respectively. From the topics people were talking about shown in Table 2, we can see that more and more residents started to blame Trump for this severe situation using hashtags #TrumpVirus and #Trumpandemic. From the online news, we can know that Trump did not take coronavirus seriously when the outbreak of coronavirus just happened which makes people feel they are paying for his arrogance. In the meantime, the appearance of hashtags such as #Survive and #StayAtHomeSaveLives denotes that people worried their own safety more than ever. As the word cloud (e) in Figure 9 shows, some new words such as “god”, “death”, “died”, “week”, “health” and “hospital” were widely used by residents. Examples are shown as follows:

• With everything that is going on with the Corona virus. I was glad that my friend decided to check up on me and my family last night. We need all of the love and support at this moment. – Satisfied relatedness need
• Trump downplayed the corona virus for months. Called it the latest democrat hoax, said we have 15 cases that will soon be 0, how can you possibly defend this obvious mismanagement? -Dissatisfied relatedness need
• Good Morning! Its Monday, Rise and Grind! Don’t let that bitch Corona stop you from accomplishing something today. Big or small goal, today YOU got it! (Cute kitten to make monday better) #MotivationalQuotes #Covid-19. -Satisfied autonomy need
• I just spent a literal hour, calling different agencies. Local and state, trying to find a place where I can report a potential corona virus violation of the Governors order to do my part as a civilian and have it looked into and got nowhere. -Dissatisfied autonomy need
• This Corona Virus is just a setback I’m still striving to achieve my goals for this year. – Satisfied competence need
• The CDC has been warning us about a future pandemic we were unprepared for. Trump cut funding to CDC and scientific research into diseases in the past and while Corona Virus is not Trumps doing, his policies made it harder for us now. -Dissatisfied competence need

### C. Overall Human Needs Analysis of the COVID-19 Pandemic in New York State

**FIGURE 10.**
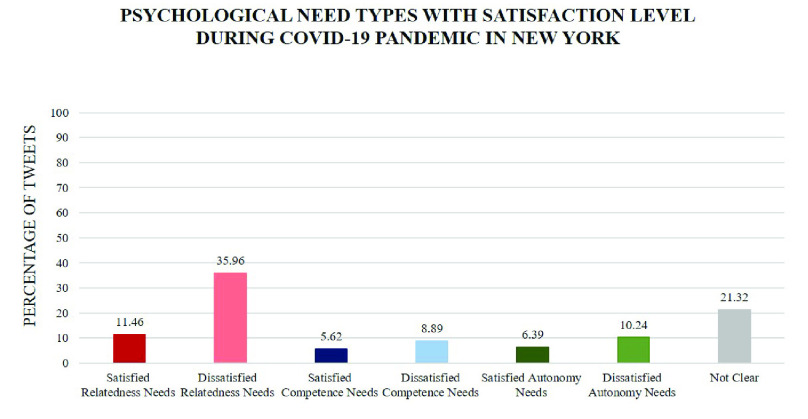
Recognizing human needs distribution during the COVID-19 pandemic in New York State using NeedFull platform.

**FIGURE 11.**
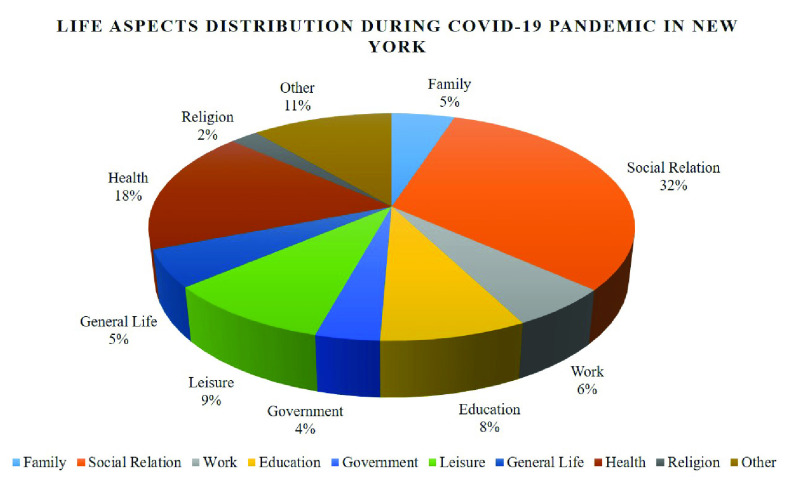
Recognizing life aspects distribution during the COVID-19 pandemic in New York State using NeedFull platform.

The analysis result shows that, throughout this period, the most pronounced human need in these tweets is relatedness with 47.42%, followed by autonomy with 16.63% and competence with 14.51%. As can be seen from Figure 10, in general, the percentages of tweets expressing frustration are larger than those of tweets expressing satisfaction for each psychological need. For example, there are 35.96% of tweets revealing dissatisfied relatedness need while the percentage of satisfied relatedness is 11.46%. Figure 7 and Figure 8 demonstrate how the need satisfaction level changed weekly. Figure 11 shows the percentages of various aspects of an individual’s life that people talked about concerning COVID-19. It is easy to see that the most pronounced life aspect is social relation with 32%, followed by health (18%), leisure (9%) and education (8%). It denotes people were eager to connect with their family and friends and they were worried about their health and education in the meanwhile. Examples for each life aspect are shown as follows:

• My mom is now scared of the Corona virus in NY so no vacation for me cause I’m not about to go alone! – Family
• We have many difference of opinions, even with our own friends, but when it comes to a common enemy, we have to put our difference of opinions aside and join our hands to fight together to extinct deadliest Virus than #Corona & save future. #Maridhas #Rajinikanth. – Social Relation
• I might go to work even if I get the corona virus. – Work
• My school just cancelled classes for next week due to the Corona Virus. #CoronaOutbreak. – Education
• Where can I get a free corona test Mr. President because your director of the CDC says I can get one but I’ve made several phone calls and I can’t get one. – Government
• i don’t know how i feel about planning a trip to vegas in may during this whole corona virus thing. – Leisure
• Can’t wait till this corona virus is over so I can stop washing my hands every time I go to the bathroom. – Health
• All my friends. My birthday was Tuesday March the 10th, but I’m extending because of the Corona virus, so I’m accepting presents over the next two weeks until the 25th of March, just wanted to let everybody know. – General Life
• Everyone please pray for my family. My mom got the corona virus. – Religion

**FIGURE 12.**
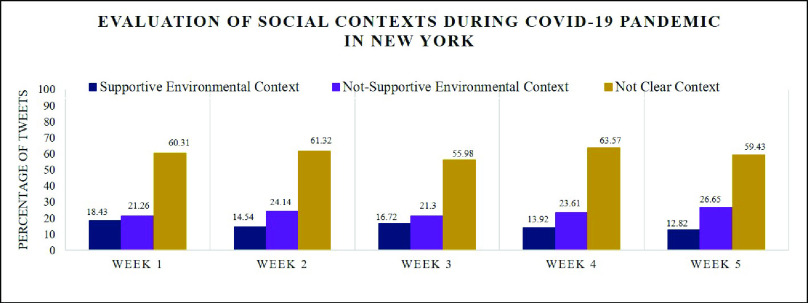
Identifying social context types during the COVID-19 pandemic in New York State using NeedFull platform.

For social context analysis, Figure 12 illustrates how people in New York State evaluate their surrounding environment regarding the satisfaction level of their psychological need during this period. In general, the percentages of unsupportive social context are larger than those of supportive social context for every week and the difference between supportive social context (12.82%) and unsupportive social context (26.65%) reached the largest number in week 5. This confirms that people’s general need were not satisfied over this period and the frustration level reached the highest in the last week shown in Figure 7 and Figure 8. As Figure 12 shows, there was a slight recovery in week 3 with 16.72% of the social context being supportive. This affirms our statement that buying essential items and getting ready for staying at home for a long period eased people’s anxiety and made people have a better evaluation of social context. Here are some tweet examples:

• My group chat is having a very detailed conversation about the corona virus, I’m glad I’m friends with smart people.-Supportive social context
• FOR CORONA VIRUS stay home make yourself happy and do something I’m cooking every day good and healthy food, and I share to help you.The best time to learn anything for your self is now FOR CORONA VIRUS.-Supportive social context
• If my last semester at college is me taking online classes because of corona virus I will go into a depression.-Unsupportive social context
• My job really sent me home because they think I have corona virus I’m dead.-Unsupportive social context

## V. Conclusion

In this paper, we propose NeedFull, an interactive and scalable tweet analysis platform, which mainly consists of four main parts: data collection module, data storage module, data analysis module and data visualization module. The four parts interact with each other and provide users with a thorough human needs analysis based on their queries. This awareness of people’s affects is a crucial step for governments and municipalities to understand their citizens’ psychological needs especially in critical times and dangerous situations. However, the human need detection model we employed can only analyze text contents. That is also the reason we cannot extend our platform to Instagram where people only share images. For future work, we plan to extend the platform with human needs analysis of other social media contents such as image and video.
